# Suppression of Core 1 Gal-Transferase Is Associated with Reduction of TF and Reciprocal Increase of Tn, sialyl-Tn and Core 3 Glycans in Human Colon Cancer Cells

**DOI:** 10.1371/journal.pone.0059792

**Published:** 2013-03-25

**Authors:** Hannah Barrow, Benjamin Tam, Carrie A. Duckworth, Jonathan M. Rhodes, Lu-Gang Yu

**Affiliations:** Department of Gastroenterology, Institute of Translational Medicine, University of Liverpool, Liverpool, United Kingdom; Universidade de São Paulo, Brazil

## Abstract

It has long been presumed, though with surprisingly little evidence, a competition between Core 1 Gal-transferase (C1GalT), Core 3 GlcNAc-transferase (C3GnT) and sialyl-transferase (ST6GalNAc-T) for elongation of O-linked mucin-type glycans initiated with GalNAcα-Ser/Thr. This study tested this presumption by selective suppression of one of these glycosyltransferases and then analysed the expressions of the enzymatic products of the other three glycosyltransferases. It was found that siRNA suppression of C1GalT markedly reduced the expression of Galβ1,3GalNAcα- (Core 1) and in the meantime increased the expressions of sialyl-GalNAcα- (sialyl-Tn), GalNAcα- (Tn) and GlcNAcβ1,3GalNAcα- (Core 3)-associated glycans in human colon cancer HT29 and SW620 cells. This supports a competitive modification of the GalNAcα-Ser/Thr between C1GalT, C3GnT and ST6GalNAc-T in O-glycan biosynthesis. As Tn, TF and sialyl-Tn are oncofetal antigens and are over-expressed in most human cancers, this information is useful for the development of glycosyltransferase-targeted therapeutic strategies for cancer treatment.

## Introduction

The biosynthesis of *O*-linked mucin type glycans is a multi-stepped, sequential, post-translational process catalysed by the expressions and activities of an array of glycosyltransferases. The biosynthesis process starts with the addition of *N*-acetyl-galactosamine (GalNAc) to the serine (Ser) or threonine (Thr) residues of the fully folded/assembled proteins to form the initial O-linked GalNAcα-Ser/Thr structure (Tn antigen) catalysed by one or two of a large family of up to 20 distinct UDP-N-acetyl-α-D-galactosamine polypeptide GalNAc-transferases (ppGalNAc-Ts) [1], [2]. These ppGalNAc-T isoenzymes have different, but partly overlapping, peptide specificities to proteins at different Ser and Thr sites and are differentially expressed in cells and tissues during development, differentiation and disease conditions such as cancer [3], [4], [5]. This first step of biosynthesis for O-linked mucin type glycans is believed to start in an inter ER-Golgi compartment [6], [7], [8] and finish in the Golgi apparatus [9], [10].

Following the formation of GalNAcα-Ser/Thr, the GalNAc residue can be modified with a Gal residue catalyzed by the Core 1 Gal-transferase (C1GalT) [11] for the formation of the Core 1 structure, Galβ1,3GalNAcα- [Thomsen-Friedenreich (TF) antigen]. The GalNAc residue of GalNAcα-Ser/Thr can also be modified with a GlcNAc residue catalysed by the Core 3 GlcNAc-transferase (C3GnT) for the formation of the Core 3 structure of GlcNAcβ1,3GalNAcα-. The GalNAc residue of GalNAcα-Ser/Thr can also be modified with a sialic acid residue by a sialyl-transferase (ST6GalNAc-T) to form sialic acid-β1,6GalNAcα- (sialyl-Tn) antigen [12], [13]. ST6GalNAc-I is believed to be the predominate sialyl-transferase for the formation of sialyl-Tn [13]. The formation of sialyl-Tn terminates the sugar chain whilst the TF and Core 3 structures can be further acted on by other glycosyltransferases in a stepwise fashion to yield up to 8 core complex glycosylation structures [14]. The core 1 to 3 glycan structures can also be modified by acetylation, fucosylation, sialylation or sulphation.

It has long been speculated that the C1GalT, C3GnT and ST6GalNAc-T compete to modify the GalNAc residue of the newly-synthesised GalNAcα-Ser/Thr for the formation of TF, Core 3 or sialy-Tn structures in living cells [15], [16], [17]. However, direct evidence that supports this competitive modification of GalNA-modification is surprisingly lacking. Mutation or inactivation of Cosmc, an ER-localized molecular chaperone that is required for the enzyme activity of C1GalT [18], has been shown to be associated with the Tn syndrome, a rare autoimmune disease in which subpopulations of the blood cells carry the incompletely glycosylated Tn antigen [19]. Treatment of human cancer cells with the O-glycosylation inhibitor Benzyl-GalNAc, a competitive inhibitor for C1GalT transferase and alpha-2,3-sialyltransferase, decreases the expression of cellular sialic acids and increases the expression of TF [20].

In this study, we assessed the consequence of selective suppression of the C1GalT by siRNA on expressions of TF, Tn, sialyl-Tn and Core 3-associated glycans in human colon cancer cells.

## Materials and Methods

### Materials

siRNA constructs against C1GalT and scrambled control non-targeting siRNA were obtained from Dharmcon (Perbio Science, Northumberland, UK). Biotinylated- *Griffonia simplicifolia* lectin II (GSL-II) was purchased from Vector laboratories (Peterborough, UK). Monoclonal antibodies against Tn (clone HB-Tn1) and sialyl-Tn (clone HB-STn1) were purchased from Dako (Pathology Products, Ely, UK). FITC-conjugated peanut agglutinin (FITC-PNA) was obtained from Sigma.

### Cell Lines

Human colon cancer HT29 and SW620 cells were obtained from the European Cell culture Collection at the Public Health Laboratory, Porton Down Wiltshire, UK and cultured in DMEM supplemented with 10% FCS, 100 U/ml penicillin, 100 µg/ml streptomycin and 4 mM glutamine as previously described [21].

### Suppression of C1GalT Expression by siRNA

The cells were cultured in triplicates in 96-well plates (5.0×10^3^ cells/well) in anti-biotic free DMEM containing 5% FCS at 37°C for 24 hr before incubated with or without 100 nM siRNA against C1GalT or control scrambled non-targeting siRNA at 37°C for 48 hr. The cells were washed and lysed for protein quantification and slot blots.

### Slot Blotting

The cellular protein extracts were blotted to nitrocellulose membrane with PR600 SlotBlot (Hoeffer Scientific Instruments, CA). The blots were blocked with 5% BSA, 0.5% tween-20 in PBS at 4°C overnight before application of monoclonal antibodies against TF (TF5) (0.2 µg/ml) [22], Tn (0.2 µg/ml), sialyl-Tn (0.03 µg/ml) or biotinylated GSL-II (0.6 µg/ml) for 1 hr. After washing and subsequent application of peroxidase-conjugated secondary antibody (3 ng/ml) or peroxidase-Extravidin (Sigma, 1∶10,000 dilution) for 1 hr, the blots were washed and visualized using a chemiluminescence Super-signal immunoblotting detection kit (Pierce; Rockford IL, USA). Densitometry analysis of the blots was performed using Image Lab software (Bio-Rad, Hemel Hempstead, UK).

### Fluorescence Immunohistochemistry

SW620 cells were cultured in 8-well chamber slides (BD Biosciences) (1×10^4^ cells/well) in anti-biotic free DMEM containing 5% FCS at 37°C for 24 hr before incubation with or without 100 nM siRNA against C1GalT or control scrambled non-targeting siRNA at 37°C for 48 hr. The cells were fixed in 2% paraformaldehyde for 10 min. After two washes with PBS, the cells were incubated with 10% rabbit serum for 1 hr before application of antibodies against STn, Tn (both 1/100 dilution in 10% rabbit serum), FITC-PNA (5 µg/ml) or biotinylated GSL-II (5 µg/ml) for 2 hr. The cells were washed with PBS and applied with FITC-conjugated secondary antibody (1∶100 dilution) or FITC-streptavidin (1∶1,000 dilution) for 1 hr. The cells were washed with PBS, mounted with DAPI-containing fluorescence mounting medium and imaged with an Olympus B51 fluorescence microscope using a 40x objective.

## Results and Discussion

**Figure 1:**
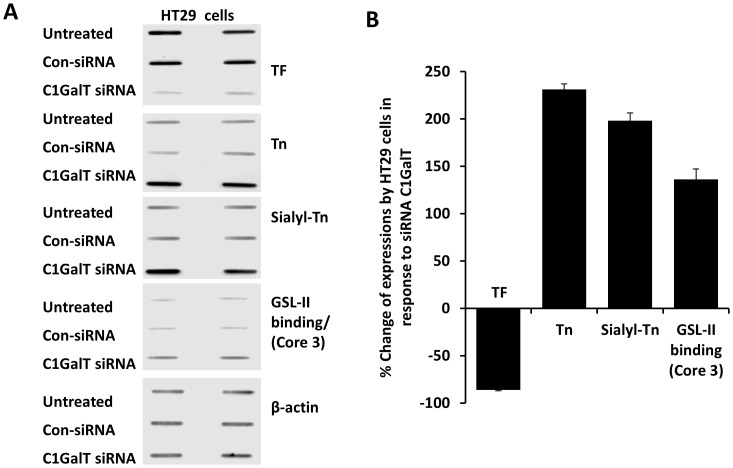
Effect of siRNA suppression of C1GalT on expressions of the cellular TF, Tn, sialyl-Tn and Core 3 glycans in HT29 cells. **A**: HT29 glycan expression in cell response to C1GalT siRNA or control siRNA. After treatment of the cells with C1GalT siRNA or control non-targeting siRNA (con-siRNA), cellular expressions of TF, Tn, sialyl-Tn and GSL-II binding (GlcNAc-, Core 3-associated glycans) were assessed by slot blots with monoclonal antibodies against TF (TF5), Tn (HB-Tn1), sialy-Tn (HB-STn1) or with biotin-GSL-II. Parallel blots were probed with antibody against β-actin for equal protein loading. Duplicate assessments are shown for each blot. **B**: Quantification of the expressions of cellular TF, Tn, sialy-Tn and GSL-II binding (GlcNAc-, Core 3-associated glycans) in HT29 cell response to C1GalT siRNA. Densities of the slots blots were quantified* and the glycan expressions are expressed as percentage change to the non-siRNA control after normalization with protein loading. *The blot densities of TF expression from untreated and C1GalT siRNA treated HT29 cells were 5004 and 715; Tn 1314 and 4345; sialyl-Tn 1634 and 4868; GSL-II binding (Core 3) 489 and 1156 and tublin 1898 and 1928.

**Figure 2:**
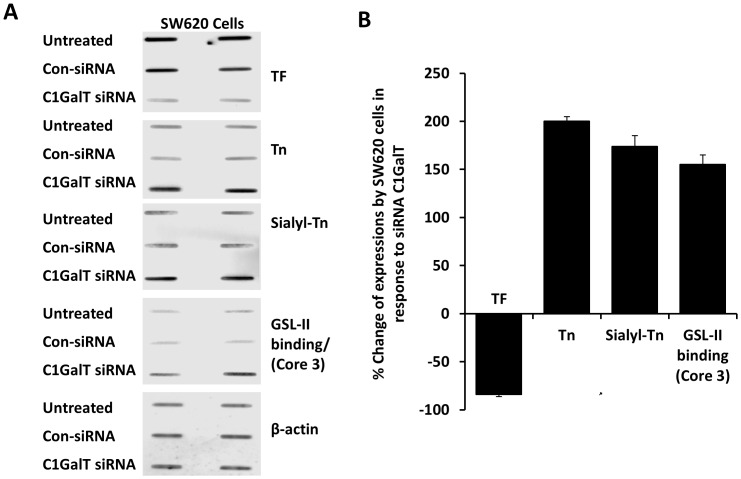
Effect of siRNA suppression of C1GalT on expressions of the cellular TF, Tn, sialyl-Tn and Core 3 glycans in SW620 cells. **A**: SW620 glycan expression in cell response to C1GalT siRNA or control siRNA. After treatment of the cells with C1GalT siRNA or control non-targeting siRNA (con-siRNA), cellular expressions of TF, Tn, sialyl-Tn and GSL-II binding (GlcNAc-, Core 3-associated glycans) were assessed by slot blots with monoclonal antibodies against TF, Tn, sialy-Tn or with biotin-GSL-II. Parallel blots were probed with antibody against β-actin for equal protein loading. Duplicate assessments are shown for each blot. **B**: Quantification of the expressions of cellular TF, Tn, sialy-Tn and GSL-II binding (GlcNAc-, Core 3-associated glycans) in SW620 cell response to siRNA C1GalT. Densities of the slots blots were quantified* and the glycan expressions are expressed as percentage change to the non-siRNA control after normalization with protein loading. *The blot densities of TF expression from untreated and C1GalT siRNA treated SW620 cells were 5144 and 834; Tn 1437 and 4313; sialyl-Tn 1748 and 4792; GSL-II binding (Core 3) 481 and 1229 and tublin 1984 and 1982.

Suppression of the C1GalT was achieved by siRNA treatment of human colon cancer HT29 and SW620 cells. The efficiency of C1GalT knock-down was monitored by cellular expression of TF with anti-TF antibody. C1GalT siRNA treatment of HT29 cells for 48 hr caused effective suppression of C1Gal1T expression as manifested by 86±3% (mean ± SD) reduction of cellular TF expression (Fig. 1 A and B). A similar reduction of the TF expression was also observed in SW620 cells after C1GalT siRNA treatment (Fig. 2 A and B).

Having effectively suppressed the C1GalT expression, we then compared the cellular expressions of sialyl-Tn (STn), Tn and Core 3 glycans. The expressions of cellular sialyl-Tn and Tn glycans were assessed by slot blots with antibodies against sialy-Tn and Tn. No antibody against Core 3 glycan is currently available and we therefore used the *Griffonia simplicifolia* lectin II (GSL-II) binding as an indicator of the expression of Core 3-associated glycans. GSL-II is a lectin isolated from *Griffonia (Bandeiraea) simplicifolia* and recognizes α- and β-linked GlcNAc residues on the non-reducing terminal of all oligosaccharides [23]. It was found that suppression of C1GalT resulted in 198±8% increase of sialyl-Tn and 136±24% increase of GSL-II binding (GlcNA-, Core 3), respectively, in HT29 and 174±11% and 155±37% increase in SW620 cells (Fig. 1 and Fig. 2). Suppression of C1GalT was also seen to be accompanied by a marked increase of Tn expression in HT29 (231±6%) and SW620 (200±5%) cells.

**Figure 3:**
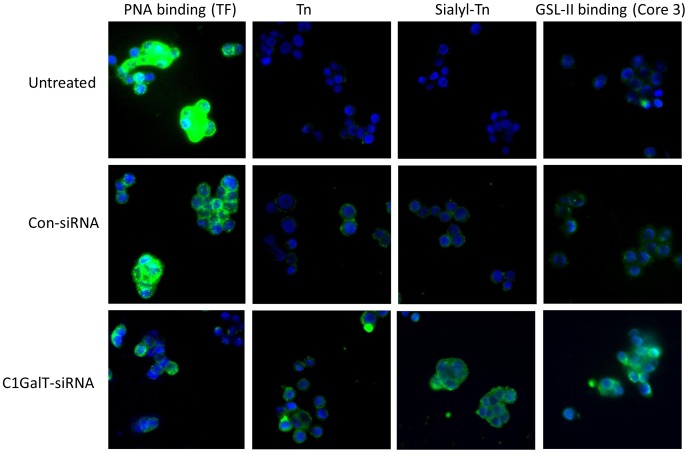
Effect of C1GalT siRNA on cellular TF, Tn, sialyl-Tn and Core 3 glycan expressions in SW620 cells. Sub-confluent SW620 cells cultured in 8-well glass chamber slides were treated without or with C1GalT siRNA or control non-targeting siRNA for 48 hr before the expressions of cell TF, Tn, sialyl-Tn and GSL-II binding (Core 3-associated) glycans were assessed by fluorescence immunohistochemistry using biotin-PNA, biotin-GSL-II or antibodies against Tn (HB-Tn1) or sialyl-Tn (HB-STn1). Representative images are shown.

To confirm these glycosylation changes observed by slot blot, we further analysed the expressions of these glycans in SW620 cells in their response to C1GalT siRNA by immunohistochemstry. Treatment of the cells with C1GalT siRNA again showed clear reduction of cellular TF expression (PNA binding) and marked increase of Tn, sialyl-Tn and Core 3 (GSL-II binding) expressions whilst treatment of the cells with control siRNA showed little effect on the expressions of these glycans (Fig. 3).

**Figure 4:**
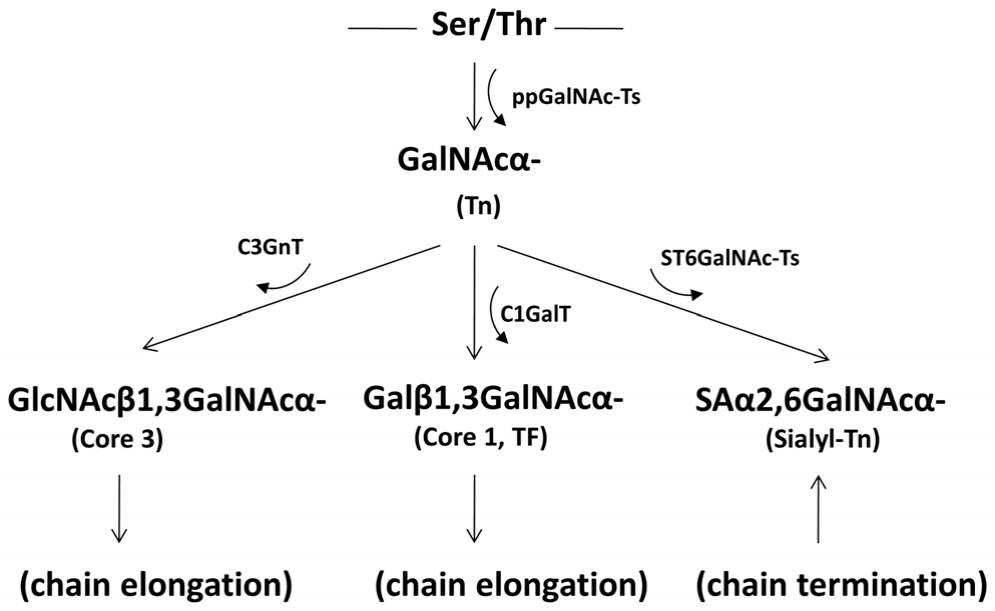
Initiation and elongation of the mucin type O-linked glycans supported by this study.

These results demonstrate that suppression of the C1GalT that controls the biosynthesis of the Core 1 structure of mucin type O-linked glycans is accompanied by increased expressions of sialyl-Tn and GSL-II binding (Core 3) in human colon cancer cells. This supports the long-suspected competitive modification of the GalNAc residue of GalNAcα-Ser/Thr between C1GalT, C3GnT and ST6GalNAc-T in the biosynthesis of complex O-linked mucin type glycans. A schematic diagram of the initiation and elongation of the mucin type O-linked glycans, supported by this study, is shown in Figure 4.

Suppression of the C1GalT was also seen in this study to result in marked increase of cellular Tn expression. This indicates that the ultimate formation of cellular TF, Tn, sialyl-Tn and Core 3 glycans are controlled not solely by the activity of these competitive glycosyltransferases. The concentrations of nucleotide sugar-donor and the rate of substrate transport throughout the Golgi have been shown previously to contribute to the expressions of specific glycans [17]. The relative positioning of the glycosyltransferases within the Golgi is also reported to be an important determinant. Work by Kellokompu and colleagues [24], [25] and by ourselves [26] has shown that Golgi derangement occurs in epithelial cancers and can be mimicked by agents that block normal Golgi acidification, in both cases leading to increased formation of oncofetal carbohydrate antigens. Furthermore, the expression and action of ER-localized molecular chaperones can also play a role in the expression of the oncofetal glycans by controlling the folding and hence the activity of the relevant glycosyltransferases [18]. Thus, the overall cellular expressions of Tn, sialy-Tn, TF and Core 3 structures are the consequence of a range of complex factors that include competition between the relevant glycosyltransferases, the spatial arrangement of the glycosyltransferases within the Golgi, the availability of nucleotide sugar-donors in the Golgi apparatus and actions of relevant molecular chaperones.

The Tn, TF and sialyl-Tn antigens are all known as oncofetal carbohydrate structures. They are expressed in fetal epithelia then become concealed by other sugar residues in healthy adult tissue but reoccur in cancerous and pre-cancerous dysplastic cells. It is estimated that up to 90% of all human cancers carry these oncofetal carbohydrate antigens [27], [28], [29], [30]. Increased occurrence of these oncofetal carbohydrate structures is associated with the development and progression of various human cancers including breast [31], colon [27], [32] and pancreatic [33] cancers. Increasing evidence suggests that alteration of these oncofetal glycans may play an active role in metastasis. Deletion of intestinal Core 1-derived O-glycans has recently been shown to cause spontaneous colitis in mice [34]. Down-regulation of C3GnT6 expression is associated with increased dysplasia/neoplasia in human colorectal cancer [35]. Over expression of sialyl-Tn antigen by cancer cells has shown to cause more aggressive cell behaviours such as increased adhesion to extra-cellular matrix and increased migration and invasion *in vitro* [36], [37] and *in vivo* in severe combined immunodeficiency (SCID) mice [37]. Overexpression of ST6GalNAc-I has shown to be co-localized with sialyl-Tn in human intestinal metaplasia as well as in gastric carcinoma and has been suggested to play an important role in sialyl-Tn overexpression in cancer conditions [12], [13]. An increased interaction between TF expressed on cancer-associated mucin protein MUC1 and circulating galectins, as a result of the increased expression of TF-expressing MUC1 by cancer cells and also of the increased release of galectins by cancer/stromal/immune tissue/cells into the circulation, both of which are common features in cancer, has been shown to promote cancer cell metastatic spread to remote organs [21], [38], [39]. This effect of the TF/MUC1-galectin interaction occurs as a result of the increased cancer cell heterotypic adhesion to vascular endothelium [38] and also as a result of cancer cell homotypic aggregation to form micro-tumour emboli that prolong tumour cell survival in the circulation and allow lodging within capillaries at the metastatic site [40], [41]. It has also been reported that breast cancer patients with higher levels of anti–TF antibody show better prognosis than the patients with lower anti-TF levels [42]. Targeting these oncofetal glycans by immunotherapy with TF-mimicking peptides for potential cancer treatment has shown promising results in mice [43].

Thus, the competition between glycosyltransferases for modification of the GalNAc residue of GalNAcα-Ser/Thr and its consequences for the expression of oncofetal carbohydrate antigens implies a potentially-useful strategy for the development of glycosyltransferase-targeted therapies for cancer.
